# Sick-Headaches

**Published:** 1874-02

**Authors:** 


Sick Headaches.—Dr. R. H. McKay, of Tennessee, writes us: For the benefit of Drs. Anstie, Wilks, and others, who suffer with headache, I ask you to publish the following formula, the efficacy of which I have tested time and again: ℞. Granulated muriate of ammonia, one teaspoonful; acetate morphia, 1 gr.; water, half pint. Dose for an adult, two teaspoonsful every ten minutes (precisely) till relief is obtained.—*Medical and Surgical Reporter.*

